# Assessment of transmission in areas of uncertain endemicity for lymphatic filariasis in Brazil

**DOI:** 10.1371/journal.pntd.0007836

**Published:** 2019-11-25

**Authors:** Amanda Xavier, Heloize Oliveira, Ana Aguiar-Santos, Walter Barbosa Júnior, Ellyda da Silva, Cynthia Braga, Cristine Bonfim, Zulma Medeiros

**Affiliations:** 1 Programa de Pós-graduação em Ciências da Saúde, Universidade de Pernambuco, Recife, Brazil; 2 Agência Pernambucana de Vigilância Sanitária, Secretaria de Saúde do Estado de Pernambuco, Recife, Brazil; 3 Departamento de Parasitologia, Instituto Aggeu Magalhães, Fundação Oswaldo Cruz, Recife, Brazil; 4 Diretoria de Pesquisas Sociais, Fundação Joaquim Nabuco, Ministério da Educação, Recife, Brazil; 5 Programa de Pós-graduação em Saúde Coletiva, Universidade Federal de Pernambuco, Recife, Brazil; Washington University School of Medicine, UNITED STATES

## Abstract

**Background:**

The objective of the Global Program to Eliminate Lymphatic Filariasis (GPELF) is to phase out this endemic disease as a public health problem by 2020. Validation of elimination is obtained from the World Health Organization through evidence of non-transmission in countries that have already been subjected to mass drug administration (MDA) and in places adjoining these endemic areas. While three municipalities in Brazil have completed MDA, the epidemiological situation remains uncertain in nine adjoining municipalities. To determine the epidemiological status, this study was to perform a review of the literature and a school-based survey to describe the past and recent endemicity of lymphatic filariasis (LF) theses nine municipalities in Brazil.

**Methodology/Principle findings:**

For review of the literature, both formal and informal literature sources were accessed since the first reports of filariasis in the Metropolitan Region of Recife, Brazil. We conducted a school-based survey in 2016 using immunochromatographic card tests (ICTs) among schoolchildren aged 6–10 years living in nine municipalities contiguous with the endemic areas in which MDA was conducted. Our review of the literature identified eight studies involving surveys demonstrating that microfilariae had been circulating in eight of the municipalities since 1967, with a low prevalence of microfilaremia, isolated autochthonous cases, and treatment of individual cases. The school-based survey included 17,222 children in 185 urban schools in the nine areas of Brazil with uncertain endemicity. One child affected by allochthonous transmission was antigen positive based on ICT and lived in a municipality adjacent to Recife; this child’s family came from Recife, but no other case was diagnosed within the family.

**Conclusions/Significance:**

The study results suggest that there is no transmission of LF in the municipalities investigated. However, these areas have population migration and socioenvironmental conditions favorable to mosquito breeding grounds; therefore, surveillance is strongly recommended in these areas.

## Author summary

Lymphatic filariasis is a parasitic disease that can cause incapacity and chronic complications. The World Health Organization aims to eliminate lymphatic filariasis as a public health problem worldwide by 2020. Four municipalities in the Metropolitan Region of Recife (Brazil) are endemic, of which three have undergone mass drug administration (MDA). However, the epidemiological status of filariasis in nine adjoining areas is unknown. Therefore, a literature review was conducted to identify reports of the vector and human cases in these nine municipalities. A review of data starting in 1967 from eight scientific reports highlighted the circulation of microfilaremia, infection of *Culex quinquefasciatus* with *Wuchereria bancrofti*, and morbidity owing to lymphatic filariasis. However, no surveillance was proposed in these areas. Therefore, in 2016, a survey of 17,222 children aged 6–10 years and enrolled in urban schools in the nine municipalities was conducted. One child from a municipality in which MDA was conducted tested positive for lymphatic filariasis, but no other case was diagnosed in her family. Our results showed no transmission of lymphatic filariasis in these nine municipalities. However, population migration and socioenvironmental conditions that favor mosquito breeding grounds indicate the need for surveillance in these areas.

## Introduction

Lymphatic filariasis (LF) is a neglected tropical disease [1]. Currently, 790 million people are at a risk of filariasis, 68 million are infected, and further 20 million experience chronic morbidity owing to this disease [2]. The objective of the Global Program to Eliminate Lymphatic Filariasis (GPELF) is to phase out this endemic disease as a public health problem by 2020. The GPELF is based on two lines of action: reduction of the prevalence of infection and management of morbidity to prevent incapacity [3–5].

In Brazil, LF is a parasitic disease caused by *Wuchereria bancrofti* and transmitted by the *Culex quinquefasciatus* mosquito vector. This disease is limited to urban areas, and only four municipalities in the Metropolitan Region of Recife -State of Pernambuco are endemic: Recife, Olinda, Jaboatão dos Guararapes, and Paulista [6,7]. Annual mass drug administration (MDA) with isolated diethylcarbamazine (DEC) 6 mg/kg for people aged ≥5 years was implemented in 2003–2017 in three of these endemic municipalities [8]; Paulista was not included owing to its low endemicity as determined using the thick drop test. Control actions have been restricted to the individual treatment of microfilaremia cases detected by health clinic surveillance activities [9].

Nine other municipalities (Abreu e Lima, Cabo de Santo Agostinho, Camaragibe, Igarassu, Ilha de Itamaracá, Ipojuca, Itapissuma, Moreno, and São Lourenço da Mata) are part of the Metropolitan Region of Recife and are areas that adjoin the endemic sites [10]. Evidence in these municipalities is limited to historical data based on case reports [6,11–17]; thus, the epidemiology of LF in these areas is uncertain [18].

Pernambuco has conducted the Transmission Assessment Survey (TAS) since 2013, which examines primary schoolchildren for the presence of LF antigenemia in each municipality following the cessation of MDA. Recife and Olinda completed three TASs with two-year intervals in 2018, while Jaboatão dos Guararapes will complete theirs in 2020 [1,8].

Consequently, surveillance and verification of the interruption of transmission started with molecular xenomonitoring (identification of parasite DNA in vector mosquitoes) and thick drop tests in two endemic municipalities of the Metropolitan region of Recife after three TASs were stopped. This included data to compile a dossier seeking validation of the elimination of LF from the World Health Organization [1,4,19]. A country is validated for elimination of LF as a public health problem if 1) it has demonstrated reduction in the prevalence of infection in endemic areas below a target threshold at which further transmission is considered unlikely even in the absence of MDA and 2) it ensures the availability of the minimum package of care for lymphoedema and hydrocele to alleviate suffering caused by the disease [19,20].

This study aimed to perform a review of the literature and a school-based survey to describe the past and recent endemicity of LF in the nine municipalities adjacent to four endemic areas.

## Methods

### Review of the literature

**Fig 1:**
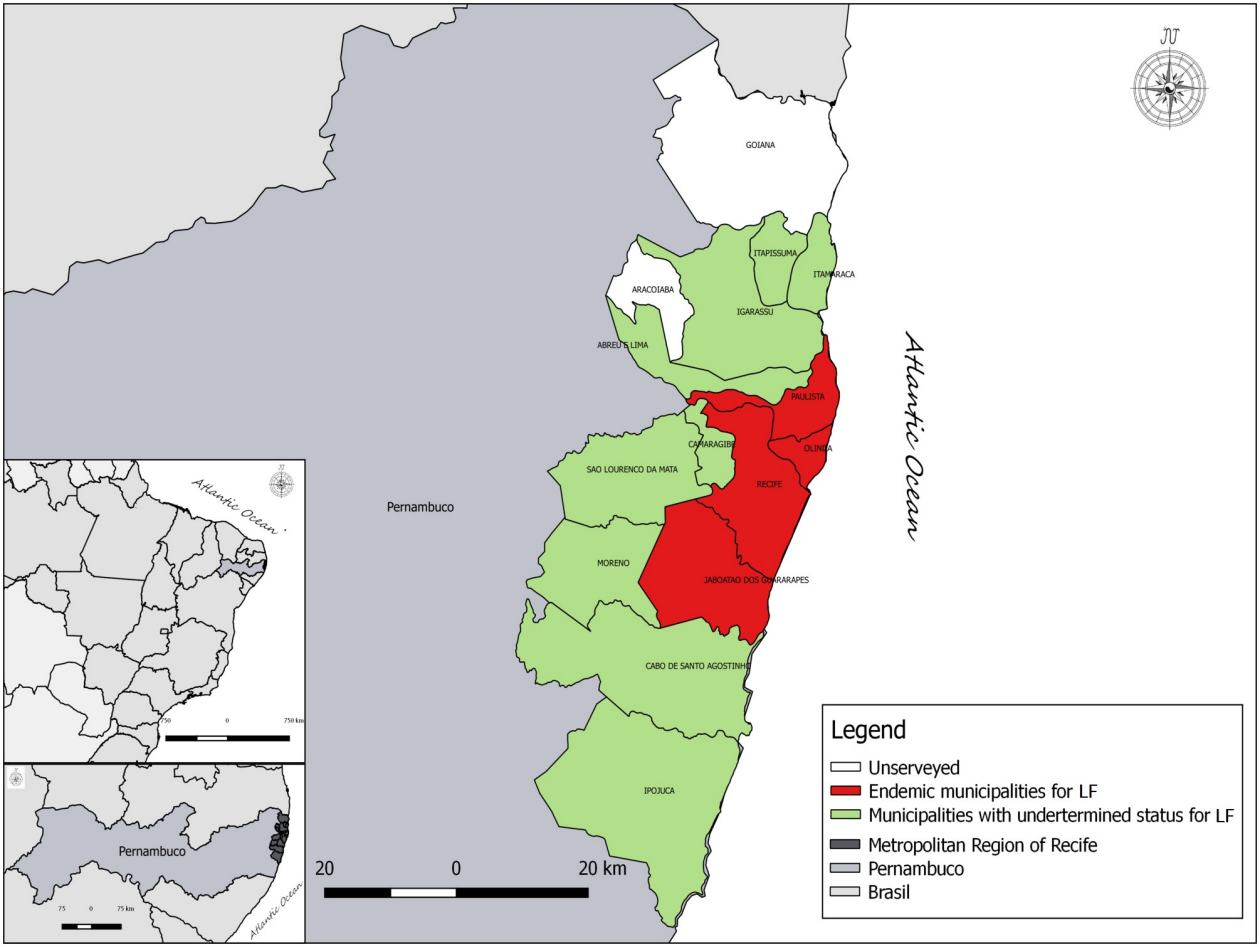
Territorial map and demographic information for the research areas. Municipalities with uncertain and endemic areas for lymphatic filariasis.

A review of the literature was conducted to determine the prevalence of LF infection. Data were identified from searches of formal and informal literature (technical reports, congressional proceedings, and non-indexed printed papers) related to nine municipalities in the State of Pernambuco that adjoin the endemic locations [8]: Abreu e Lima, Cabo de Santo Agostinho, Camaragibe, Igarassu, Ilha de Itamaracá, Ipojuca, Itapissuma, Moreno, and São Lourenço da Mata (Fig 1).

The LILACS, SciELO, PubMed, and Medline databases were searched for articles published between January 1, 1967 and December 31, 2017 using the keyword “Brazil” combined with “Epidemiology”, “Lymphatic filariasis”, and “Health Surveys”. Retrieved articles were supplemented by analyzing their bibliographic references, informal congressional abstracts, and data from the Brazilian Federal Health Department (published reports and documents).

### School-based survey of LF

#### Survey strategies

This descriptive and observational study used the STrengthening the Reporting of OBservational studies in Epidemiology (STROBE) checklist in accordance with the STROBE guidelines [21] (S1 Checklist).

### Ethical considerations

LF surveys were conducted as a public health activity. For the children included in these surveys, written consent from at least one parent or guardian, along with assent from the child, was required. Approval for the study was obtained from the research ethics committee of the Instituto Aggeu Magalhães, Fundação Oswaldo Cruz (CAEE 07392812.6.0000.5190).

### Survey areas and populations

The study included nine municipalities in the State of Pernambuco, located in Northeastern Brazil, that geographically adjoin the municipalities endemic for LF [8] (Fig 1). We enrolled children aged 6–10 years (compared to those aged 6–7 years recommended for TAS) [22] from all municipal public schools in the urban area to increase the chances of detecting infected individuals. The children included in the survey lived close to the schools, and their name, age, sex, and address were collected from school records.

The sample size calculation of the schoolchildren in each municipality was performed based on the following population parameters: filarial antigenemia prevalence of 50% (unknown), design effect of 1.0, standard error ranging from 1.5 to 2.5%, and 95% confidence interval. The formula used was: *n* = [EDFF×Np(1-p)]/ [(d^2^/Z^2^_1-α/2_×(N-1)+p×(1-p)], where *n* = population size (for finite population correction factor [fcp]), *p* = frequency % hypothetical of factor results in the population, *d* = limit confidence interval % of 100 (absolute +/-%), and *EDFF* = effect size (for group survey). The assumed loss of subjects because of operational difficulties owing to the technique used, which involves blood collection, was an estimated 20%. The random sample of the schoolchildren in each municipality was obtained from the list of students provided by the schools. More details can be found in supplemental document 2 (S1 Table).

### Diagnostic tools and data collection

Immunochromatographic card tests (ICTs) were used to test for circulating filarial antigen (BinaxNOW Filariasis, Alere Scarborough, Orlando, United States). For these tests, 100 μL of capillary blood was collected. If the results were positive, the circulating microfilariae were investigated and quantified using the polycarbonate membrane filtration technique with 3-μm-sized pores [23], and ultrasound examinations of the cervical, axillary, and inguinal lymph node chains, and scrotum were performed to identify nests of adult worms [24–26]. All family members of children with positive test results were also examined according to the protocol using these diagnostic tools.

### Data entry and analysis

Epi Info version 7.2 was used for data analysis. The children’s homes were georeferenced by identifying geographic coordinates using Google Earth. If the addresses could not be located, QGIS 2.18 software was used, which continued the street database for the Metropolitan Region of Recife available by the Instituto Brasileiro de Geografia e Estatística or the Global Position System was used in combination with a local visit. Based on the coordinates, a kernel estimator was used to produce population density mapping on the constructed maps. The critical cut-off was determined by SSB software and varied by district [22].

## Results

### Review of the literature

**Fig 2:**
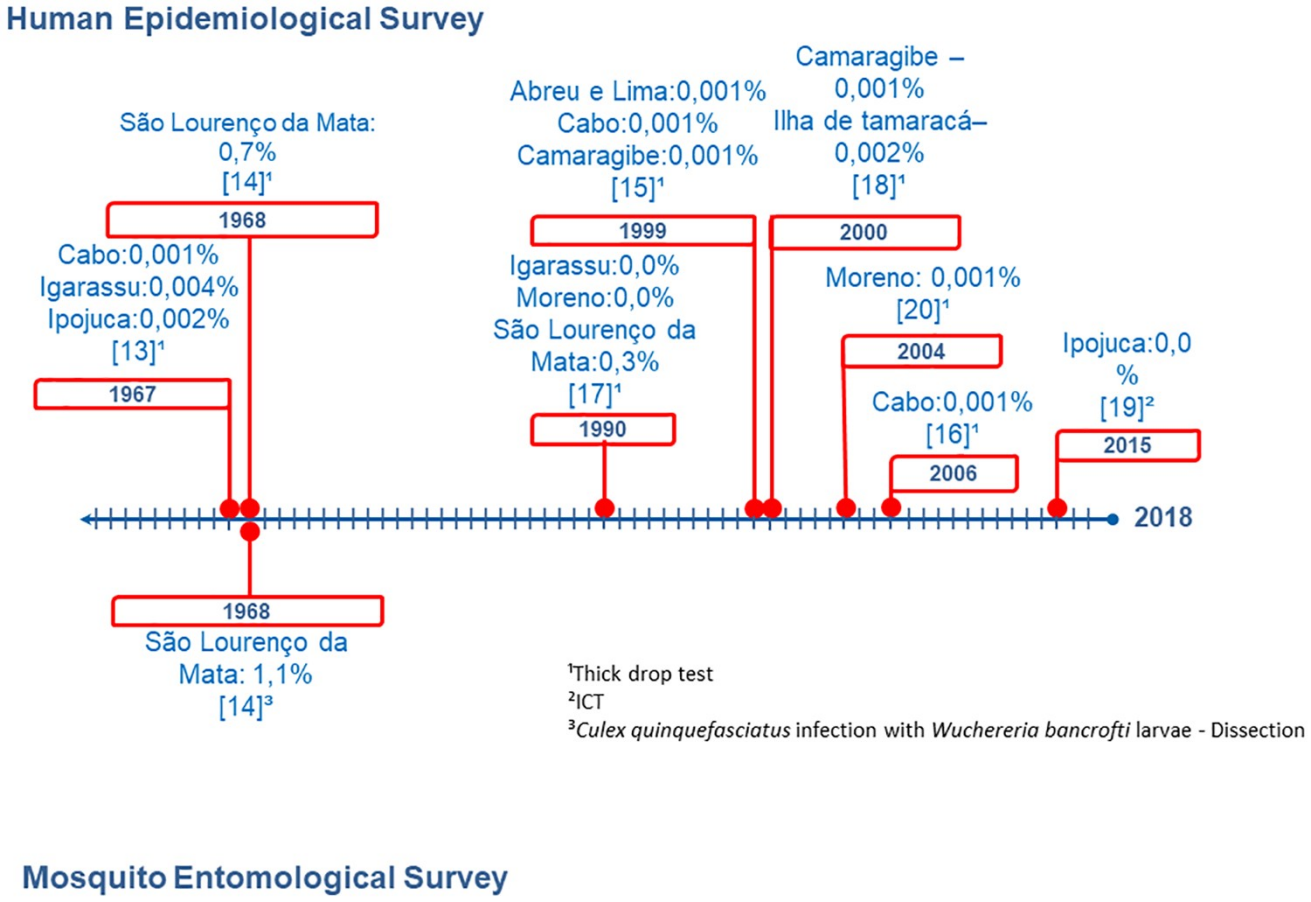
Landmarks in the evolution of epidemiology and entomological surveys in Pernambuco.

The review of the literature identified eight epidemiological surveys performed during the study period. Six of them used thick drop tests to identify microfilaria in the blood, one used the thick drop test and investigated filarial larvae in mosquitoes, and one investigated antigens using ICTs. There was no record of any survey in the municipality of Itapissuma. Fig 2 shows that in the remaining eight municipalities, there were cases of microfilaremia, corresponding for the prevalence of <1% [11–15]. The vector infection rate was 1.1% in a single survey conducted in the municipality of São Lourenço da Mata in 1967 [12]. In 2015, an investigation of antigens conducted in the municipality of Ipojuca did not identify any cases of LF [16]. In all studies, all microfilaremia cases were treated with DEC (6 mg/kg/12 days).

Table 1 shows the reports of autochthonous cases in the municipalities of Abreu e Lima [13], Cabo de Santo Agostinho [13, 14], Camaragibe [13], and Ilha de Itamaracá [6]. Cases of filarial morbidity were detected in Moreno [17] and Cabo de Santo Agostinho [14]. The allochthonous cases identified in Cabo de Santo Agostinho [14], Camaragibe [13], Ilha de Itamaracá [6], and Moreno [17] were from Recife, Jaboatão dos Guararapes, and Olinda.

**Table 1:** Autochthonous cases, filarial morbidity cases, and vector tests from municipalities in Pernambuco.

Surveys			Human					Vector		Morbidity			
References	Population	Methods	No. tests		No. infected			No. tests (house/female mosquitoes)	% infected	ADLA**	Hydrocele	Cloudy urine	Elephantiasis
			Thick drop	ICT	Autochthonous	Allochthonous	NI***						
**Abreu e Lima**													
[13]	Soldiers >18 years	Prevalence study	23,773*		01¹								
**Cabo de Santo Agostinho**													
[11]	General population	Prevalence study	2,829				03						
[13]	General population	Prevalence study	23,773*		01^2^								
[14]	General population	Prevalence study	7,650		01³	05^5^				80	20	02	07
**Camaragibe**													
[6]	General population	Prevalence study	1,554			02^6^							
[13]		Prevalence study	23,773*		04^4^								
**Igarassu**													
[11]	General population	Prevalence study	3,164				12						
[15]	General population	Prevalence study	72		--	--	--						
**Ilha de Itamaracá**													
[6]	General population	Prevalence study			05	08^7^							
**Ipojuca**													
[11]	General population	Prevalence study	2,358				05						
[16]	General population	Prevalence study		960	--	--	--						
**Moreno**													
[15]	General population	Prevalence study	54		--	--	--						
[17]	General population	Prevalence study	2,513			02^8^				43	15	--	01
**São Lourenço da Mata**													
[12]	General population and female *Culex quinquefasciatus*	Prevalence study	2,459				17	356/ 754	1.1				
[15]	General population	Prevalence study	1,985				05						

*total research sample, independent of the municipality;

** ADLA = acute dermatolymphangioadenitis;

*** NI- No information; Autochthonous—subject who always lived in the same neighborhood. 1- Caetes neighborhood; 2—Ponte dos Carvalhos district; 3—Pontezinha district 4- neighborhood: 2 from Fabrica, 1 case from dos Estados and 1 from Bairro Novo; 5- from Recife and Jaboatão dos Guararapes; 6- from Recife; 7- from Recife, Jaboatão dos Guararapes and Olinda; 8- from Recife

### School-based survey of LF

**Fig 3:**
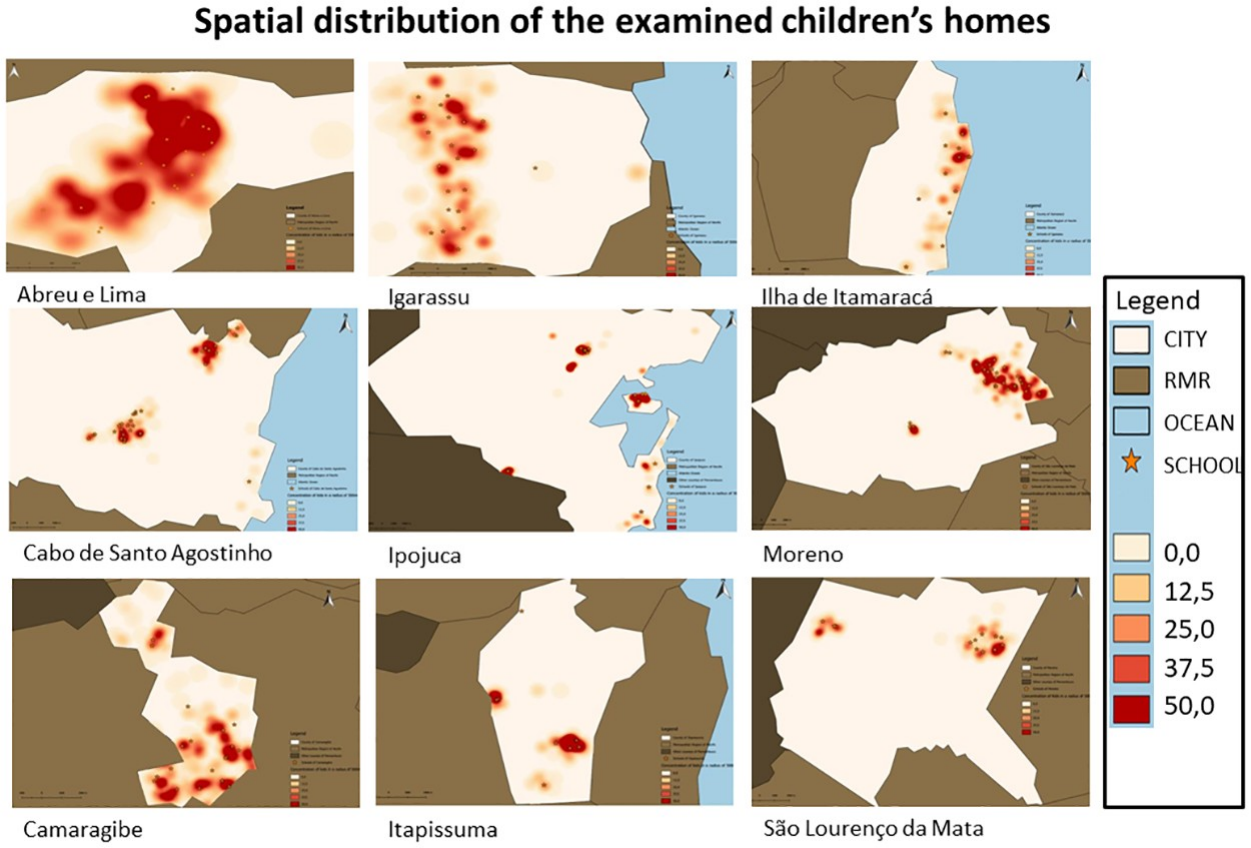
Spatial distributions of the examined schools. The spatial distributions of the schools included in the survey were homogeneous in all urban areas of Abreu e Lima, Cabo de Santo Agostinho, Camaragibe, Igarassu, Ilha de Itamaracá, Ipojuca, Itapissuma, Moreno, and São Lourenço da Mata.

A total of 17,222 children who were enrolled in the 185 public schools in the nine municipalities were surveyed. Female subjects predominated (51.25%), and the mean age was 8.16 years (SD 0.03). The location of the children’s homes (Fig 3) showed a homogenous spatial distribution in the urban areas of the nine municipalities. A kernel estimator made it possible to identify agglomerates of schoolchildren within each municipality, with concentrations in urban areas (Fig 3).

Only one child in the municipality of Camaragibe was positive for the presence of circulating filarial antigen (Table 2). This child was a six-year-old girl from the Nova Descoberta district of the municipality of Recife (a post-MDA area). The child was born in Recife and had moved to Camaragibe with her family in 2014. She was amicrofilaremic, and ultrasound examination did not reveal any adult worm nests in the cervical, axillary, or inguinal lymphatic chains. Investigations of parasite, antigens, and adult worms in other members of her family (parents and three siblings) were negative. However, her parents had undergone three rounds of MDA while living in Recife.

**Table 2:** Distribution of schoolchildren in the municipalities participating in the survey, according to age, sex, and test results, 2016.

Districts		Abreu e Lima	Cabo de Santo Agostinho	Camaragibe	Igarassu	Ilha de Itamaracá	Ipojuca	Itapissuma	Moreno	São Lourenço da Mata
Results										
**Pop. aged 6–10 years (2010 census)**		8,134	15,400	12,283	8,458	1,492	7,100	2,206	4,756	9,328
**No. of urban schools** **tested**		22	32	25	24	13	21	09	13	26
**Sample** **size**		1,767	3,138	2,870	1,387	666	3,176	833	974	2,411
**Age (years)**	**6–7 (95% CI)**	597–33,79% (31,58–36,00)	946–30,15% (28.54–31.76)	993–34,60% (32.86–36.34)	578–41,67% (39.08–44.26)	167–25,08% (21.79–28.37)	1,270–39,99% (38.29–41.69)	272 (32,65%) (29.47–35.83)	267 (27,41%) (24.61–30.21)	1,017 (42,18%) (40.21–44.15)
	**8–10 (95% CI)**	1,170–66,21% (64.00–68.42)	2,192–69,85% (68.24–71.46)	1,877–65,40% (63.66–67.14)	809–58,33% (55.74–60.92)	499–74,92% (71.63–78.21)	1,906–60,01% (58.31–61.71)	561 (67,35%) (64.17–70.53)	707 (72,59%) (69.79–75.39)	1,394 (57,82%) (55.85–59.79)
**Sex**	**Female (95% CI)**	864–48,90% (46.57–51.23)	1,632–52,00% (50.25–53.75)	1,471–51,25% (49.42–53.08	698–50,32% (47.69–52.95)	340–51,05% (47.25–54.85)	1,625–53,37% (51.64–55.10)	399 (47,89%) (44.50–51.28)	490 (50,30%) (47.16–53.44)	1,307 (54,20%) (52.21–56.19)
	**Male (95% CI)**	903–51,10% (48.77–53.43)	1,506–48,00% (46.25–49.75)	1,399–48,75% (46.92–50.58)	690–49,68% (47.05–52.31)	326–48,05% (44.26–51.84)	1,551–48,83% (47.09–50.57)	432 (52,11%) (48.72–55.50)	484 (49,70%) (46.56–52.84)	1,104 (45,80%) (43.81–47.79)
**Critical Cut-off**		09	09	09	09	06	09	07	08	09
**No. of ICT** **+ves**		00 (0,00%)	00 (0,00%)	01 (0,00%)	00 (0,00%)	00 (0,00%)	00 (0,00%)	00 (0,00%)	00 (0,00%)	00 (0,00%)

CI—Confidence interval

ICT—Immunochromatographic card test

## Discussion

Validation of the elimination of LF as a public health problem requires the assessment of transmission interruption through a detailed review of historical and epidemiological evidence [4]. The present report provides the first school-based survey of LF endemicity in areas in Brazil with uncertain infection status. A review of the literature on the historical prevalence of this disease was conducted, along with a survey to identify the prevalence of LF in urban areas of nine municipalities adjoining the endemic areas in the State of Pernambuco Abreu e Lima, Cabo de Santo Agostinho, Camaragibe, Igarassu, Ilha de Itamaracá, Ipojuca, Itapissuma, Moreno, and São Lourenço da Mata.

The results of the review of the literature revealed that since 1967, microfilaremia has been circulating in eight of these municipalities, with low prevalence and isolated autochthonous cases [11]. In 1968, one study evaluated the infection rate in mosquitoes in São Lourenço da Mata; however, no population survey or surveillance was performed by the health facility. [12]. Our review showed that positive cases were treated individually using DEC, and based on these results suggesting a low LF prevalence, none of the nine municipalities met the epidemiological criteria for MDA [9]. Two articles from 2004 [17] and 2006 [14] reported on LF morbidity. The prevalence of LF manifestations can be used for epidemiological mapping of filarial disease [27,28]. A study conducted in Jaboatão dos Guararapes, in an area adjoining these two municipalities, identified a very strong association between hydrocele and filariasis infection, showing that the observation of this clinical manifestation provides a reliable means of rapidly diagnosing LF, thus assessing endemic areas [29].

It is possible that places adjoining endemic areas might have both low endemicity and cases of morbidity. The allochthonous cases identified in the study were from Recife, Olinda, and Jaboatão dos Guararapes. Regions adjoining endemic areas that have undergone MDA should have disease notification systems. Moreover, these places require healthcare services that can make differential diagnoses of LF-associated morbidity and deliver treatment [30].

No protocol has been defined within the GPELF for evaluating areas with uncertain endemicity [31]; therefore, the present study enrolled children aged 6–10 years in these municipalities with no prevalence data. The original protocol (TAS) [22] was not applicable owing to the uncertainty regarding the prevalence of LF in these areas; thus, MDA was not required. The ICT survey of schoolchildren was conducted in the same way as that conducted in studies in Tanzania and Ethiopia [32] and Bangladesh [33]. In those studies, this model was used in areas adjacent to endemic areas, irrespective of whether or not MDA had been implemented. In Bangladesh, the evaluated area was similar to that of our study based on historical data [33]. However, the studies in Tanzania and Ethiopia [32] used the methodology underlying the confirmatory mapping tool; therefore, they included schoolchildren aged >7 years to improve the chance of detecting infected individuals. In areas where MDA had been implemented, individuals aged 6–7 years were supposedly protected against filarial infection because they were born after the interruption of transmission via this treatment [32] and because the migratory flow of individuals at this age was low [34].

ICT was chosen because it is practical and easy to applyand has been shown to be sensitive and specific [33] in areas that are endemic only for *W*. *bancrofti* [29–36]. In the present study, this tool was used to diagnose a case of filariasis in which all tests (investigations of microfilaria and adult worms) were negative. Filarial investigations of all family members were negative, and the family members came from an area in the district of Nova Descoberta, Recife, where MDA had been implemented. However, the adults in this family had not participated in all five rounds of treatment. In 2017, surveillance identified other cases of microfilaremia in the same district [37]. The presence of cases is an indicator of the need for surveillance, since positive findings are predictive of the risk for the reintroduction of infection [38], suggesting that Nova Descoberta is a residual focus post-MDA.

The data from these studies reinforce the hypothesis that internal migration may be an important factor in the spread of LF [34]; the results of the present study highlight the fact that there were no barriers around the municipalities and that vectors were present. Moreover, precarious socioenvironmental situations [39] favor the transmission of this endemic into disease-free areas. Even if transmission is successfully interrupted through the GPELF, the continued presence of vector breeding sites increases the risk of transborder migration as a source of transmission [34]. In recent years, there has been migration of Haitians to Brazil, and cases of microfilaremia have been identified in this group. These cases could be a source for transmission of LF in disease-free areas [37,40–42]. Thus, the surveillance of migration to countries that are participants in the GPELF is important even after validation of LF elimination.

The mapping approach, which was proposed by WHO, is simple and practical and works well in high-prevalence areas. However, there are concerns regarding its reliability in low-prevalence areas in which ICTs are used, and the protocol is not clear regarding sample definition and the criteria for selecting human or vector samples [43]. The results of the present school-based analysis showed that MDA was unnecessary in these regions. Surveys of children based on ICTs are used by the GPELF to determine whether MDA should be suspended; however, they can also be used to map areas in which endemicity is uncertain. Surveillance is necessary in regions with a low prevalence that do not require MDA but are proximal to endemic areas because there is a risk of introduction of infection [32].

The data provided by previous reports described on the review of the literature, particularly the older reports, are not comparable owing to the different methodological approaches adopted. In addition, a considerable number of reports did not provide detailed information about the methods used for obtaining these data. Similar to the circulating filarial antigen survey conducted in uncertain areas, selection bias might have occurred because the present study was conducted in schoolchildren of public schools only. However, more than 70% of our study population (aged 6–10 years) was enrolled in public schools, and most of the children were from low-income families [44]. Most children were enrolled in public schools near their homes.

In countries endemic for LF, such as Brazil, in which MDA is indicated for some areas but not others, surveillance needs to be stratified according to the previous prevalence, type of vector, and environmental and demographic factors that influence its transmission. Socioenvironmental information can be used to stratify these areas according to the likelihood of transmission and in association with spatial and geostatistical analyses, it is an excellent tool to aid in surveillance [45,46]. Nonetheless, there is also a need for rigorous surveillance in areas of low endemicity that do not require MDA and that harbor precarious socioenvironmental conditions that favor the transmission of LF.

In summary, this study focused on surveys in areas in Brazil with uncertain LF endemicity. Our review of the background data showed that since 1967, microfilaremia has been circulating, infection of *C*. *quinquefasciatus* with *W*. *bancrofti* has been observed, and a burden of LF morbidity has been present. Nonetheless, no surveillance has been proposed for these areas. This study shows the utility of TAS-like methods to determine LF presence in areas of uncertain endemicity that were never treated. Moreover, it underlines the importance of surveillance in areas that have stopped MDA and in areas adjoining these previously treated areas. It is important that places with a low prevalence that have not received MDA and border areas with endemicity that have received MDA be subject to surveillance to avoid the risk of recrudescence. These areas may be determined from a combination of data on sociodemographic factors, migration, and sentinel site information, along with the capacity of the local healthcare infrastructure.

## Supporting information

S1 Checklist STROBE Checklist. (DOC)

S1 Table Calculated sample sizes of schoolchildren for each of the municipalities. (DOCX)
