# Supplementary material for: Assessment of transmission in areas of uncertain endemicity for lymphatic filariasis in Brazil
Source: PLoS Negl Trop Dis. 2019 Nov 25;13(11):e0007836. doi: 10.1371/journal.pntd.0007836 (PMC6917295; doi:10.1371/journal.pntd.0007836)
Supplement: S1 Table — (DOCX) [file pntd.0007836.s002.docx]

**S2 Table. Calculated sample sizes of schoolchildren for each of the municipalities, 2016.**

| Municipalities | Population of children aged 6–10 years* | Estimated prevalence (%) | CI | Standard error | Design effect | Sample | Sample + 20% |
| --- | --- | --- | --- | --- | --- | --- | --- |
| Abreu e Lima | 3.039 | 50 | 95% | 2.0 | 1.0 | 1.341 | 1.609 |
| Cabo de Santo Agostinho | 6.419 | 50 | 95% | 1.5 | 1.0 | 2564 | 3.073 |
| Camaragibe | 5.302 | 50 | 95% | 1.5 | 1.0 | 2365 | 2.838 |
| Igarassu | 2.557 | 50 | 95% | 2.0 | 1.0 | 1238 | 1.485 |
| Ilha de Itamaracá | 1.189 | 50 | 95% | 2.5 | 1.0 | 637 | 764 |
| Ipojuca | 5.498 |  | 95% | 1.5 | 1.0 | 2403 | 2.833 |
| Itapissuma | 1.200 | 50 | 95% | 2.3 | 1.0 | 722 | 948 |
| Moreno | 1.880 | 50 | 95% | 2.5 | 1.0 | 846 | 1.015 |
| São Lourenço | 4.803 | 50 | 95% | 2.0 | 1.0 | 1601 | 1.921 |

*All children enrolled in public municipal schools

CI – Confidence Interval;
